# Patient-Guided Breast Reconstruction Education

**DOI:** 10.7759/cureus.9070

**Published:** 2020-07-08

**Authors:** Ivo A Pestana

**Affiliations:** 1 Plastic and Reconstructive Surgery, Wake Forest Baptist Medical Center, Winston-Salem, USA

**Keywords:** patient education, breast reconstruction, educational resources

## Abstract

Background

Breast reconstructive surgeons must discuss large amounts of information in an expedient manner to a growing group of women. Our aim is to identify the breast reconstruction resources women prefer and how they desire information to be conveyed in order to develop patient-guided reconstruction education.

Methods

A preoperative and postoperative breast reconstruction survey was given to women planning to undergo breast reconstruction or who have already undergone reconstruction. The surveys asked women to rank educational resources utilized. Questions on the timing of information gathering, desired educational content, and wish to speak with other breast reconstruction women were included.

Results

One hundred and fifty consecutive women were enrolled in the study, 50 in the preoperative group and 100 in the postoperative group. Preoperatively, women wish to utilize their surgeon more than any other resource, and the postoperative survey identified that patients utilize their surgeon even more than expected (p < 0.05). Internet and pamphlets were utilized second and third most frequently. Women desired an interactive compact disc significantly more than this resource is currently being utilized (p < 0.05). There was a strong desire to speak to women who had undergone the process. Sixty-six per cent of women indicated they would attend two or more meetings to learn about breast reconstruction.

Conclusion

The reconstructive surgeon remains the most important educational resource for their patients. Providing consultation over more than one meeting, adding uncommonly discussed content (specifics on postoperative care, body image changes and expectations) to the consultation, and connecting reconstruction patients may improve preoperative consultations and satisfaction with the process of breast reconstruction.

## Introduction

The rise in breast reconstruction rates is due to multiple reasons including rising incidence of breast cancer in women, improved health insurance coverage, increasing rates of contralateral prophylactic mastectomy, and an expansion of indications for nipple-sparing mastectomy [1-3]. Surgical options for breast reconstruction have developed in tandem with the rising number of women desiring it. Prepectoral reconstruction and the myriad of autologous tissue options are just a few examples of recent developments available to women undergoing breast reconstruction [4,5]. 

In the era of patient-centered medicine, women welcome as much individualized information as can be gathered on medical topics, including breast reconstruction, and effective communication/education on a subject leads to improved healthcare outcomes [6-8]. Taken together, reconstructive surgeons face the task of providing increasing amounts of breast reconstruction information to increasing numbers of women in an effective and efficient manner. A variety of breast reconstruction educational resources are available to aid in this process. Although various educational aids exist, patients and providers report varying results with their use [9-12]. Thus, each surgeon must decide which breast reconstruction resources to employ and how to integrate them into their practice and patient population.

Our primary aim is to elucidate how, and to what degree, women currently utilize resources to learn about the breast reconstruction process. Furthermore, we aim to provide insight into the optimal timing of information delivery, length of time spent gathering information, and preferred modality of information transfer in order to develop effective patient-guided breast reconstruction counseling in this growing patient population.

## Materials and methods

Over a three-year period, two Institutional Review Board-approved questionnaires were distributed to consecutive women presenting for immediate or delayed breast reconstruction consultation (Preoperative-Reconstruction Survey) or to women who had previously undergone breast reconstruction (Postoperative-Reconstruction Survey). The format for breast reconstruction consultation consists of an initial 30- to 60-minute evaluation at our institution’s multidisciplinary breast center and includes history, physical examination, and discussion of reconstruction options specific to the patient. Women subsequently return to our departmental clinic within two to four weeks for review of the reconstructive plan as well as preoperative preparation, depending on reconstruction type planned. The follow-up visits typically are of 30-minute duration. Women who are referred from a practice outside our health system’s breast clinic have similar visits. If immediate reconstruction is planned, women will meet with our oncologic breast surgeons during these visits. 

Women included in the preoperative group had a surgical indication for mastectomy and had not previously seen a reconstructive surgeon. Women receiving prophylactic mastectomy for gene mutations or strong family history of breast cancer were also included in this group. Women excluded from the preoperative group were those that previously had a reconstructive consultation and women who had undergone a reconstructive operation. Women included in the postoperative group had previously undergone breast reconstruction (immediate or delayed reconstruction, successful or failed reconstruction). Women excluded from the postoperative group were those who had undergone mastectomy without any form of breast reconstruction. Women who filled out the study preoperatively did not necessarily fill out the study postoperatively depending on when she completed reconstruction. Women were not required to fill in every answer of the survey to be included accounting for the occurrence of questions with less than 100% response rate. Patients ranked resources utilized or resources desired on a scale of 1 (most utilized/desirable) to 5 (least utilized/desirable). Resources not ranked were assigned a value of 6.

Comparison of educational resources utilized or desired by women within the same population (Preoperative or Postoperative groups) was completed using a student’s t-test. Wilcoxon signed-rank test was employed to compare resource use between the two groups. Questionnaire response data were collected in a prospective manner and statistical significance of p less than 0.05 was utilized.

## Results

One hundred and fifty consecutive women were enrolled in the study. Fifty women were enrolled in the preoperative study group with an average age of 52.7 (±9.5) years. One hundred women were enrolled in the postoperative study group with an average age of 52.8 (±10.4) years. Race characteristics of enrolled women are demonstrated in Table 1. 

**Table 1:** Patient demographics and race characteristics

Option		Preoperative Group (50 total)		Postoperative Group (100 Total)									
Age		52.7 (±9.5)		52.8 (±10.4)									
White		37		85									
African American		10		12									
Asian		1		1									
Other		2		1									
Hispanic		4		3									
Non-Hispanic		46		97									

In the post-reconstruction group, 69 patients underwent implant-based reconstruction, while 31 patients underwent autologous reconstruction.

Women in the preoperative group identified their reconstructive surgeon to be their most desired source of information (Table 2). In addition to the reconstructive surgeon, pre-reconstruction women also indicated the desire to utilize interactive compact discs (CD) (Table 2). When evaluating the resources actually used by postoperative women, this trend continued with women indicating that they utilized their surgeon at a significantly higher rate than they indicated preoperatively (Table 3). Although women included in the preoperative group did not necessarily complete the postoperative questionnaire, women in the postoperative group identified their surgeon as the most desired education resource and reported they did not desire the use of an audio resource or interactive CD as much as the preoperative women did (Table 2).

**Table 2:** Preoperative educational resources desired versus postoperative educational resources utilized Patients ranked resources utilized or resources desired on a scale of 1 (most utilized/desirable) to 5 (least utilized/desirable). Resources not ranked were assigned a value of 6.

Option	Preoperative Group Desired	Postoperative Group Utilized	p-value
Pamphlet/Booklet	3.1	3.6	0.13
Mobile Phone App	4.7	5.2	0.06
Interactive Website	3.1	3.3	0.5
Audio	4.9	5.6	<0.0001
Interactive CD	4.7	5.5	<0.0001
Surgeon	1.7	1.2	0.006

**Table 3:** Breast reconstruction educational resources utilized versus desired by postoperatively-surveyed women Patients ranked resources utilized or resources desired on a scale of 1 (most utilized/desirable) to 5 (least utilized/desirable). Resources not ranked were assigned a value of 6.

Option	Utilized	Desired	p-value
Pamphlet/Booklet	3.6	3.7	0.48
Mobile Phone App	5.2	5.2	0.93
Interactive Website	3.3	3.4	0.06
Audio	5.6	5.3	0.01
Interactive CD	5.5	4.9	0.0003
Surgeon	1.2	3	<0.00001

Among the post-reconstruction group, the resources utilized and desired did not show a statistical difference when comparing autologous and prosthetic-based reconstruction. However, women who underwent autologous reconstruction trended toward utilizing interactive websites at a higher rate and desired the use of an interactive CD more than women who underwent implant-based breast reconstruction (Table 4).

**Table 4:** Influence of reconstruction technique on breast reconstruction educational resources utilized versus desired by postoperatively surveyed women Patients ranked resources utilized or resources desired on a scale of 1 (most utilized/desirable) to 5 (least utilized/desirable). Resources not ranked were assigned a value of 6.

	Resources Utilized		Resources Desired	
Option	Implant	Autologous	Implant	Autologous
Pamphlet/Booklet	3.5	3.8	3.6	3.9
Mobile Phone App	5.1	5.4	5.1	5.3
Interactive Website	3.6	2.8	3.3	3.5
Audio	5.6	5.7	5.3	5.2
Interactive CD	5.5	5.4	5.1	4.4
Surgeon	1.2	1.1	2.8	3.5

To determine if the educational style or discussion presented by the specific surgeon affected educational resource use, analyses of patient survey answers specific to the attending physician who cared for them were completed. These comparisons reveal no significant differences between resources desired or resources utilized (Table 5).

**Table 5:** Postoperative survey response results based on attending (ATTG) surgeon Patients ranked resources utilized or resources desired on a scale of 1 (most utilized/desirable) to 5 (least utilized/desirable). Resources not ranked were assigned a value of 6.

	ATTG 1	ATTG 2	ATTG 3	ATTG 4	ATTG 5	ATTG 6
RESOURCES UTILIZED						
Pamphlet/Booklet	3.7	3.3	4	3	3	3.7
Mobile Phone App	5.4	5	5.2	5	5	5.2
Interactive Website	3.2	3.4	3	2.2	4.8	3.7
Audio	5.6	5.7	5.6	5.2	5.3	5.7
Interactive CD	5.3	5.5	5.8	4.2	5.5	5.6
Surgeon	1	1.3	1.2	1	1	1.3
RESOURCES DESIRED						
Pamphlet/Booklet	3.8	3.3	3.3	4.4	5	3.9
Mobile Phone App	5.3	5.3	4.9	5.2	6	4.9
Interactive Website	3.3	3.3	3.4	3.2	5.8	3.1
Audio	5.1	5.1	5.2	5.6	5.3	5.7
Interactive CD	4.7	4.5	4.7	4.6	5.5	5.6
Surgeon	2.9	2.8	3.1	4.2	3.5	2.9

When assessing the patients by age group, women age 60 or younger were more likely to utilize an interactive website than those who were age 61 or older (p < 0.05).

Several trends were identified when comparing Caucasian and non-Caucasian patients. Preoperatively, Caucasian patients indicated that they would like to utilize a mobile phone application and speak with their surgeon at a higher rate than non-Caucasian patients. Postoperatively, there was a tendency for Caucasian women to desire interactive websites more than non-Caucasian women. There was no difference in resources actually utilized (Table 6).

**Table 6:** Breast reconstruction educational resources utilized or desired by race Patients ranked resources utilized or resources desired on a scale of 1 (most utilized/desirable) to 5 (least utilized/desirable). Resources not ranked were assigned a value of 6.

	White	Non-White	p-value
Preoperative Resources Desired			
Pamphlet/Booklet	2.94	3.54	0.3
Mobile Phone App	4.49	5.46	0.04
Interactive Website	2.92	3.62	0.24
Audio	4.92	4.69	0.59
Interactive CD	4.67	4.69	0.97
Surgeon	1.41	2.31	0.05
Postoperative Resources Utilized			
Pamphlet/Booklet	3.52	3.87	0.49
Mobile Phone App	5.21	5.07	0.7
Interactive Website	3.24	3.8	0.28
Audio	5.58	5.8	0.35
Interactive CD	5.44	5.6	0.55
Surgeon	1.18	1.13	0.81
Postoperative Resources Desired			
Pamphlet/Booklet	3.85	2.87	0.08
Mobile Phone App	5.18	5.2	0.95
Interactive Website	3.21	4.33	0.05
Audio	5.18	5.67	0.19
Interactive CD	4.84	5.2	0.43
Surgeon	3.05	2.8	0.7

The timing of providing breast reconstruction information to women was assessed. Preoperatively, 32% (16/50) of women stated they would like to receive the information prior to meeting with their reconstructive surgeon and 66% (33/50) stated that they would like to receive the information at the same time as meeting with their reconstructive surgeon. Of those women who wanted to receive the information at the time of their consultation, 42% (14/33) stated that they would like to review the information alone and 58% (19/33) stated that they would like to review the information with their surgeon.

In assessing how long this information should take to review, 62% (31/50) of women indicated that this information should take less than 30 minutes to review, while 84% (42/50) indicated that it should take one hour or less. Seventy per cent of women (35/50) were willing to attend more than one meeting to learn about breast reconstruction, with 50% (25/50) willing to attend two meetings, 16% (8/50) willing to attend three meetings, and only 4% (2/50) with a desire to pursue more than three meetings. A single meeting was desired by 26% of women (13/50).

Women who had previously undergone reconstruction were asked about the timing of their reconstructive surgeon consultation. Fifty-seven per cent of post-reconstruction women indicated that they would like to meet with their reconstructive surgeon on the same day or shortly after meeting with their oncologic surgeon, while 35% of women indicated that they would like to meet with their surgeon in a delayed fashion. Only a minority (3%) of women indicated that they would have postponed their meeting with the reconstructive surgeon until after surgical extirpation of their tumor.

The majority of both pre- and post-reconstruction women viewed speaking with other women who had undergone reconstruction as an important part of the reconstruction education process. When asked preoperatively, 66% (33/50) of these women indicated that they would like to speak with women regarding breast reconstruction. Interestingly, approximately half (16/33) of these patients were interested in speaking with both women who had previously undergone reconstruction and those who had not. Postoperatively, 53% of women indicated that they spoke with other women and found it very helpful, while an additional 29% of women indicated that they would have liked the opportunity to do so. Only 15% of women did not have the desire to speak with other women, while 4% did speak with other women and did not find it helpful. When asked how much they would be interested in speaking with other women about their experience, the post-reconstruction women responded in a very positive light, with an average response of 7.9 out of 10.

The top five pieces of information pre-reconstruction women wanted to learn about during their reconstruction consultation were: recovery time after surgery, duration of the entire process of breast reconstruction, specifics of surgical techniques, postoperative instructions or restrictions, and body image following surgery. The top five questions that post-reconstruction women indicated they wished they had asked before undergoing breast reconstruction were: details of immediate postoperative care, care following the initial healing of wounds, pain related to tissue expansion, body image and appearance to expect, and a general desire for more information about the process of reconstruction.

## Discussion

The challenge faced by the breast reconstructive surgeon lies not only in what information to provide, but how, when, and in what format and with which aids the counseling is provided. As the landscape of the physician-patient relationship has evolved to a patient-centered approach over the past decades, this topic has received even more focus [7,13].

Heller and Miller outline the three most important questions facing women considering breast reconstruction as to whether or not to have reconstruction, when to have reconstruction, and how this reconstruction should be performed [14]. The role of the physician in these decisions can be vastly different and can also depend on the decision-making pattern of the patient [15,16]. Perhaps, the most important finding of our study is that most patients continue to view their physician; in this case, their reconstructive surgeon, as the primary source of information and support to make these decisions. In a time when information is increasingly available through other avenues, the value of this observation cannot be overstated. It is well documented that the way information is given by healthcare providers strongly influences patient preferences [17,18]. As surgical specialists with an ever-expanding role in the treatment of breast cancer, it is important to evaluate not just the information we provide to patients, but also the manner in which that information is provided. Utilizing effective communication has been proven in meta-analyses to improve patient outcomes [19].

With a myriad of modalities for transferring information in the modern world, each with its associated positives and negatives, the reconstructive surgeon must be selective in teaching aids used. Following the reconstructive surgeon, the most valued sources utilized by patients were the internet, followed by information pamphlets provided to them. The only resources women indicated they utilized significantly less than they originally intended were audio resources and an interactive CD. This observation is especially interesting considering the only resource preoperative women indicated they wished to utilize significantly more was such a CD. Over a decade ago, an interactive digital aid, administered by interactive CD, was developed and proved effective in the breast reconstruction decision-making process [10]. Despite the decline in popularity of the CD over this same time period, our survey results suggest interactive digital aids remain a desired educational resource.

With women utilizing the internet as their second most popular information resource, it is worth noting the recorded literature on the worldwide web regarding breast reconstruction. Previous studies have noted various rates of utilization of the internet as an information source. Joyce et al. noted only 33% of women indicated that the internet would be a useful form of communication, despite 63% of their patient population spending over an hour a day online and almost 50% of their patient population being aware of specific websites [20]. Losken et al. also noted a high rate of utilization of internet for reconstruction education, with 68% of women indicating they gathered information about breast reconstruction from internet-based resources. Interestingly, Losken et al. found women wish to access their surgeon via the web, with 81% of women indicating that they should be able to contact their surgeon via email [21]. Although a significant amount of women are utilizing the internet to learn about breast reconstruction, multiple studies indicate the overall quality of information available online is poor in both quality, content, and readability [22,23]. Moreover, there are differences in what healthcare providers and their patients view as important when receiving counseling [24]. Therefore, knowledge of what women who are about to embark on the reconstructive journey and those who have completed it want to know about the process is imperative. Based on our survey, the most common questions women have prior to undergoing the reconstructive process are focused on what type of postoperative care will be needed, the specifics of surgical technique used, and expectations regarding body image following surgery. These sentiments were echoed in the postoperative survey. The responses to these open-ended questions have made discussion of these topics routine during a consultation and driven the creation of a departmental breast reconstruction resource addressing these subjects. The patient reviews this resource before or after their consultation.

Interesting trends based on race were identified in our study population. Caucasian women were more likely to desire electronic resources, such as interactive websites or mobile phone applications based on our survey. Similar to disparities in access to breast reconstruction, studies have identified a disparity in access to internet resources in non-Caucasian populations, and our data may reflect this fact [25-27]. Identifying those patients who may have limited access to certain resources is crucial to providing them with the proper vehicle of information to make an informed decision. In fact, a lack of appropriately delivered resources may contribute to the disparity between the rate of breast reconstruction between Caucasian and non-Caucasian women [28]. In this context, the expansion of electronic resources may only benefit a single population of women in whom breast reconstruction is already common. Individual or more widespread focus on the information contained in pamphlets or booklets was shown in our study to be just as popular as internet resources and may be more accessible to the general population. It is this medium that the author has expanded in his practice in order to provide a more comprehensive review of the breast reconstruction process.

In addition to education resource type and content, duration and timing of the education are also critical to tailoring care to the patient. Seventy per cent of women indicated that they would be willing to attend two or more meetings to learn about breast reconstruction. Furthermore, 57% of women wished this meeting to be on the same day or the day after their meeting with the surgical oncologist, while 35% wished this to be a delayed meeting scheduled prior to their oncologic surgery. Based on these findings, scheduling multiple meetings, with one being the same day as the oncologic surgeon and one in a delayed fashion, may be a benefit in order to give women time to read the information given to them, gather their own resources, review all decision aids, and assimilate this information into any questions they may have for the second meeting [29].

One of the most significant findings of our survey was the potential role of communication between women who had previously undergone breast reconstruction and women who were contemplating reconstruction. Seventy per cent of pre-reconstruction women indicated that they would like to speak with other breast reconstruction patients. Postoperatively, 81% of women indicated that they either spoke with other women, and found it helpful, or would have liked to speak with other women. With a relative value ranking of 7.9/10, women were overwhelmingly willing to share their experiences with others. Although communication between patients has previously been noted to be an important factor, the extent to which it seems to be important may have been underestimated [30]. Such an observation could create exciting new pathways for patient education. Social media platforms, online forums, and mobile phone applications combine patient connectivity with ease of use. While the safety tools are not yet in place to account for compliance with the Health Insurance Portability and Accountability Act (HIPAA), development of such a platform for patients undergoing breast reconstruction would provide an outlet for patients to share their experiences and provide vital information to each other outside of the confines of a physician’s office. On a smaller level, simply collecting the contact information from patients who wish to share their experience can provide new patients with a source of knowledge that they may not have otherwise had.

Our limitations were those of any single-center study, namely that our patient population was limited to reconstruction patients in our institution’s catchment area possibly affecting patient heterogeneity. Furthermore, there was a disparity in the number of patients enrolled in the preoperative and postoperative groups due to limited resources at the site of preoperative patient enrollment in the surgical oncology clinics. Another weakness of our study stems from the lack of inclusion of alternative methods of giving breast reconstruction patients information, such as telephone, video, and nurse specialist-led consultations. Although these options are not available at our institution, they are important adjuncts in today’s healthcare system. Data identified by our study will be a benefit to those performing these types of encounters since both physician and other healthcare providers may convey it verbally.

## Conclusions

The process of breast reconstruction education and counseling is an extremely complex one. While the best method of information transfer will always be surgeon-specific and patient-centered, consideration should be given to several aspects of this process. First and foremost, the reconstructive surgeon remains the primary educational resource for their patients. Due to this, meeting with patients on more than one occasion, including in-depth discussions about topics not commonly a focus in the initial reconstructive consultation (i.e., details of postoperative care, addressing expected body image changes through the potential multi-step reconstructive process), connecting breast reconstruction patients through HIPAA compliant means, and directing patient’s to specific, well-informed, and appropriately structured websites are likely a benefit to the counseling process. It is our hope that by incorporating this information into the process of breast reconstruction education/ counseling, each surgeon can provide the best outcomes for their patients.
